# The Role of the Polymeric Immunoglobulin Receptor and Secretory Immunoglobulins during Mucosal Infection and Immunity

**DOI:** 10.3390/v10050237

**Published:** 2018-05-03

**Authors:** Holly Turula, Christiane E. Wobus

**Affiliations:** 1 Department of Microbiology and Immunology, University of Michigan, Ann Arbor, MI 48109, USA; hturula@umich.edu; 2 Graduate Program in Immunology, University of Michigan, Ann Arbor, MI 48109, USA

**Keywords:** polymeric immunoglobulin receptor, secretory immunoglobulin, mucosa, infection

## Abstract

The gastrointestinal tract houses millions of microbes, and thus has evolved several host defense mechanisms to keep them at bay, and prevent their entry into the host. One such mucosal surface defense is the secretion of secretory immunoglobulins (SIg). Secretion of SIg depends on the polymeric immunoglobulin receptor (pIgR), which transports polymeric Ig (IgA or IgM) from the basolateral surface of the epithelium to the apical side. Upon reaching the luminal side, a portion of pIgR, called secretory component (SC) is cleaved off to release Ig, forming SIg. Through antigen-specific and non-specific binding, SIg can modulate microbial communities and pathogenic microbes via several mechanisms: agglutination and exclusion from the epithelial surface, neutralization, or via host immunity and complement activation. Given the crucial role of SIg as a microbial scavenger, some pathogens also evolved ways to modulate and utilize pIgR and SIg to facilitate infection. This review will cover the regulation of the pIgR/SIg cycle, mechanisms of SIg-mediated mucosal protection as well as pathogen utilization of SIg.

## 1. Introduction

**Figure 1:**
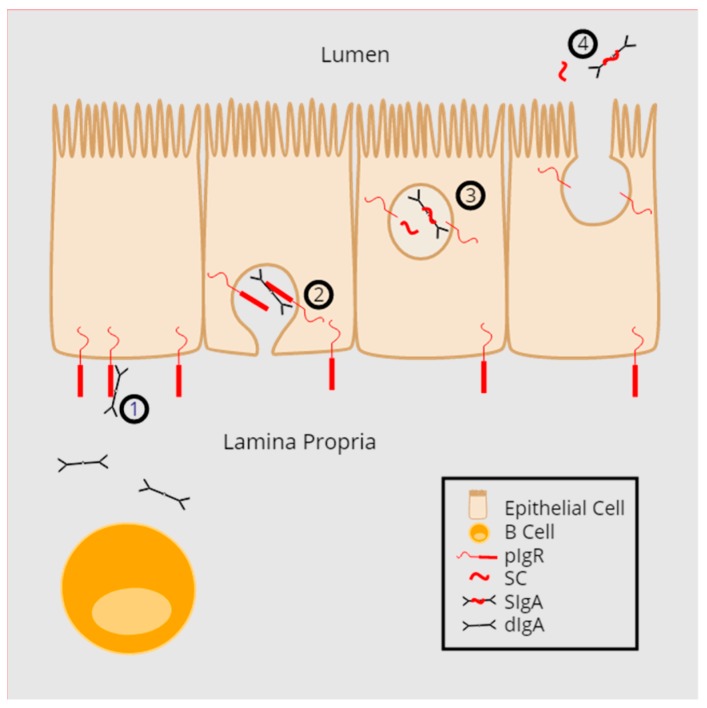
Transport of polymeric immunoglobulins (pIg) to the mucosal surface. **(1)** pIg (dimeric IgA [shown] or pentameric IgM [not shown]) made in the lamina propria bind to polymeric immunoglobulin receptor (pIgR). **(2)** Endocytosis and transcytosis of the pIg:pIgR complex from the basolateral to the apical side of the mucosal epithelium. **(3)** Intracellular proteolytic cleavage of pIgR creating secretory component (SC) and SIgA. **(4)** Release of SC and SIgA to the mucosal surface.

The host is in constant contact with millions of microbes. To protect itself from these microbes, it has developed an array of defense mechanisms. Epithelial cells connected by tight junction complexes are a critical barrier that separate the host interior from the outside world. The protective capacity of this physical barrier is further enhanced by innate and adaptive immune responses. One main immunologic mechanism at mucosal barriers is polymeric immunoglobulins (pIgs), specifically dimeric immunoglobulin A and polymeric immunoglobulin M. PIgs are made by plasma cells in the lamina propria underlying the epithelial barrier (Figure 1). They are then transported across the epithelial barrier with the help of the polymeric immunoglobulin receptor (pIgR). Secretion and release of pIgs into the luminal space occurs following proteolytic cleavage of pIgR. The pIg molecule bound to the cleaved, extracellular portion of pIgR is called secretory immunoglobulin (SIg). SIgs, of which SIgA is the most abundant, and secretory components (SC) can mediate host protection through specific and non-specific pathogen interactions. SIg and SC mediate innate protection of the host via immune exclusion, neutralization, and complement activation, but also aid in adaptive immunity by modulating immune cell activation and function, and by maintaining homeostasis. Thus, pIgR is critical for the protective function of SIgs.

SIgs in the gastrointestinal tract are polyreactive against several bacterial species and primarily target intestinal commensal bacteria [1]. Most of these “natural” anti-commensal SIg are made through T cell-independent B cell responses [2]. Despite the lack of T cell help, these natural polyreactive SIgs can bind to antigens with high affinity, sometimes equivalent to that of T cell-dependent SIgs [3]. SIgs help to shape bacterial communities by reducing their immunogenicity and by sequestration within the mucus layer [4]. SIg-immune complexes can then be sampled by the host immune system via microfold (M cells), which in turn initiate both local and systemic responses [5]. 

Herein, we will present an overview of the pIgR/SIg system and its role during infection with a focus on the importance of pIgR in the gastrointestinal tract. We will further highlight the role of pIgR/SC and SIg as a microbial scavenger capable of manipulating host immunity, and address pathogen modulation and utilization of pIgR and SIg to facilitate infection.

## 2. pIgR Structure and Function 

PIgR is a highly glycosylated, type I transmembrane protein with a predicted molecular mass of ~81 kDa that is conserved among all vertebrates [6]. The extracellular portion is composed of six domains: five immunoglobulin-like domains, and a sixth, which contains a highly conserved cleavage signal [7]. The intracellular domain contains signals for endocytosis, intracellular sorting and transcytosis. PIgR is expressed on the basolateral surface of ciliated epithelial cells in the mucosal epithelium [8]. Expression is inhibited in mucus-producing goblet cells by secretory leukocyte protease inhibitor (SLPI) via the NFkB pathway [9]. The main function of pIgR is to transport dimeric immunoglobulin A (dIgA) and polymeric immunoglobulin M (pIgM) from the lamina propria across the epithelial barrier to mucosal surfaces in four main steps (Figure 1) [10]. **1.** PIg made in the lamina propria binds non-covalently via the joining (J) chain to the extracellular domain 1 of pIgR, on the basolateral surface of the epithelial layer [11,12]. **2.** Once bound, the receptor and Ig undergo clathrin-mediated endocytosis, and are transcytosed through the epithelial cell to the mucosa [6]. **3.** Upon approaching the apical surface, the pIg bound domain of the receptor undergoes endoproteolytic cleavage, likely by a host serine proteinase [13], and disassociates from the membrane-bound domain, forming secretory component (SC). SC remains associated with pIg, forming SIg. Unbound pIgR can also be transcytosed via the endosome to the luminal side of the epithelium alongside with pIg-bound pIgR. It similarly undergoes endoproteolytic cleavage forming SC and releasing free, unbound SC. **4.** Upon release, SC and SIg diffuse into the mucus layer [14]. Therefore, pIgR plays a vital role during the generation of SIgs and becomes a part of SIgs. The clinical importance of pIgR is further underscored by the finding that multiple polymorphisms in the *PIGR* gene are linked with immunoglobulin A nephropathy [15].

## 3. The Multiple Functions of Secretory Component (SC)

SC has multiple functions beyond facilitation of pIg transport and is critically important for the function of SIg [16,17]. First, SC enhances SIg stability. While SC does not alter SIg antigen affinity [18,19], SC is thought to help SIg resist proteolytic degradation by host and bacterial enzymes in the intestinal lumen [20,21]. However, at least one pathogen has evolved ways to overcome the enhanced resistance to proteolysis. Specifically, streptococcus-specific proteases degrade pIg and SIg similarly [22]. Second, SC aids in appropriately localizing SIg in the mucus layer. Both the SC and pIg are glycosylated via N- and O-linkages [23,24]. These glycosylations aid in transcytosis and release of SIg from the epithelial cells [25]. In the distal gastrointestinal tract, SIg diffuses through the thick inner mucus layer and adheres to the outer mucus layer where intestinal bacteria are localized via binding to these carbohydrates [14]. Third, SC is a non-specific microbial scavenger. Both N- and O-linked glycosylations mediate attachment of bacteria, aiding in sequestration of bacteria in the mucus layer [26]. Thus, SC promotes intralumenal sequestration of bacteria. Fourth, SC can also neutralize the effect of toxins and prevent infections [27,28]. Fifth, SC has homeostatic functions in the epithelium. For example, it can prevent activation of neutrophil effector functions [29] and neutralize IL-8 activity [30]. Taken together, SC is a critical player in the mucosal defense arsenal. 

## 4. Regulation of the pIgR/SIg System

PIgR expression and SIg secretion are modulated by multiple factors: immunological, microbial, hormonal and environmental [31]. A main regulator of pIgR expression are immune system mediators, including interferon γ (IFNγ) and tumor necrosis factor alpha (TNFα) [7]. Regulation occurs at the transcriptional level and several transcription factor binding sites, including for nuclear factor κ-light-chain-enhancer of activated B cells (NF-kB) and interferon regulatory factor 1 (IRF1), are found near the 5′ end of the *PIGR* gene. Thus, pIgR gene transcription and subsequently pIgR:pIg transcytosis are upregulated following NF-κB activation [32]. Several immune signaling cascades, including toll-like receptor (TLR) activation and inflammatory cytokine signaling, converge on NF-κB and have been demonstrated to directly upregulate pIgR gene expression and pIgR:pIg transcytosis both in vivo [33,34] and in vitro [8,35,36]. Thus, it is not surprising that bacteria, bacterial products, and viruses also stimulate pIgR expression in vitro [33,37,38]. This was also confirmed in vivo. For example, bacterial upregulation of pIgR expression during infection was observed during *Chlamydia* infection in the epithelium of the human reproductive tract [39]. In addition, pIgR protein expression increases distally throughout the small intestine of mice, correlating with increasing concentrations of bacteria [40,41]. Hormones, such as estrogen, progesterone and androgen, are another group of host factors that regulates *PIGR* expression [17]. Thus, pIgR levels change during the estrous cycle, and pIgR is upregulated in mammary glands during lactation [42]. Furthermore, environmental factors such as diet [43], exercise [44], alcohol consumption [45], and likely smoking [46] also alter pIgR levels. For completeness, we note that recent work also indicates modulation of pIgR expression in cancer [47,48,49]. Hence, the functions of pIgR go beyond the mucosal surface. Nevertheless, most studies to date have focused on the critical role of pIgR as a key mucosal defense mediator. 

## 5. Lessons from pIgR-Deficient Mice

**Figure 2:**
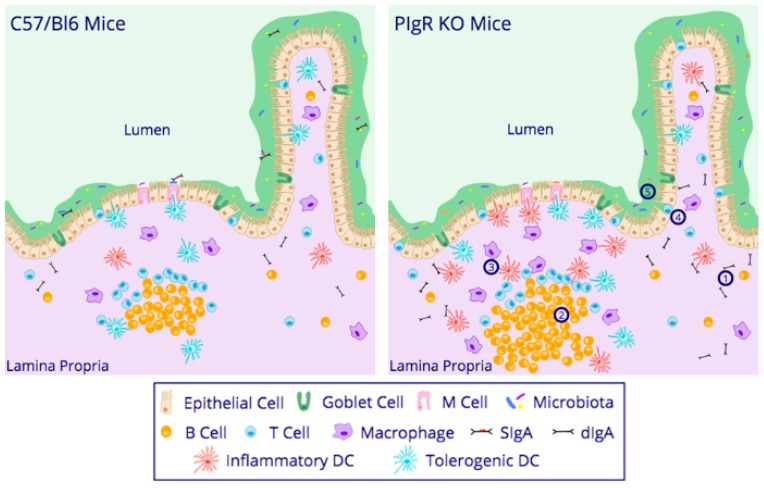
Small intestinal alterations in pIgR knock-out (KO) mice compared to C57/Bl6 control mice. PIgR KO mice exhibit enhancements in: **(1)** serum IgA, **(2)** B cells, **(3)** macrophages and dendritic cells, and **(4)** intraepithelial lymphocytes. PIgR KO mice also exhibit **(5)** alterations in bacterial communities.

In order to directly assess the role of pIgR/SIg in mucosal homeostasis, pIgR-deficient (pIgR KO) mice were generated [50]. Although there are many similarities to C57/Bl6 control mice, pIgR KO mice exhibit five key differences (Figure 2). **1.** The lack of pIgR results in a lack of secretion of dIgA into the mucosa, and a buildup of serum IgA compared to WT mice [50,51]. **2.** Serum IgA levels may be further augmented in pIgR KO mice due to elevated numbers of B cells in the lamina propria compared to controls [52,53]. **3.** PIgR KO mice also have increased dendritic cell (DC) and macrophage numbers in the Peyer’s patch compared to controls [53]. **4.** Although no differences were found in CD4 T cells of the Peyer’s patch, spleen, and mesenteric lymph nodes [52], increased quantities of small intestinal intraepithelial lymphocytes (IELs) in pIgR KO mice have been reported [54]. The latter is thought to be mediated through the enhanced differentiation of immature hematopoietic precursor cells, not because of changes in proliferative capacities, ex vivo cytotoxicity, or migration into the intestinal epithelium [54]. In the lung, lack of SIgs through pIgR depletion (pIgR KO) results in an upregulation of pulmonary natural killer cells [55]. **5.** Removal of pIgR results in alterations in the commensal microbiota. Although an initial littermate-controlled study found no alteration in bacterial communities in mice lacking PIgR [56], these findings have a caveat, namely, since SIg can pass through the digestive system [57], it may have been passed along from pIgR-sufficient littermate controls to pIgR KO mice obscuring any potential changes. Consistent with that, a subsequent, non-littermate study using 16S rRNA analysis did reveal alterations in the feces and cecal microbiota in pIgR KO vs. WT mice and suggested an overall 7% change in intestinal bacterial communities in the absence of pIgR [58]. Despite differences in microbial communities, colonic mucus thickness is similar in pIgR KO mice compare to controls [14]. Small intestinal mucus thickness has not been directly assessed; however, small intestinal permeability may increase with age in pIgR KO mice compared to controls [59]. **6.** PIgR KO mice have enhanced ileal IFNγ and iNOS levels compared to controls [53], likely because of the increases in certain immune cell subsets. Given alterations in antigen-presenting cells and inflammatory mediators in pIgR KO mice, it is hardly surprising then, that inflammatory diseases such as chronic obstructive pulmonary disease [55,60,61,62], DSS-induced and T-cell-mediated colitis in mice [58,63], as well as Crohn’s disease and ulcerative colitis in humans [64,65] are highly prevalent when SIgs are absent or reduced. Alterations to the immune baseline of the pIgR KO mice may also be beneficial or detrimental during infection. For example, enhanced susceptibility and mortality to gastrointestinal infections in pIgR KO mice was observed for *Salmonella* and *Giardia* [66,67]. However, deletion of pIgR was detrimental during primary murine norovirus infection, as viral titers in the gastrointestinal tract were reduced compared to controls [53]. Reduced infection was proposed to be due to increased intestinal anti-viral cytokine levels. Taken together, these data demonstrate the critical role for pIgR/SIgA in promoting mucosal homeostasis through mediation of cytokine production and immune cell development, and highlight its necessity in protection from inflammatory diseases.

## 6. Agglutination and Exclusion of Pathogens from Mucosal Surfaces (Immune Exclusion)

An important defense mechanism of the pIgR/SIg cycle is via agglutination of pathogens and exclusion from mucosal surfaces. This mechanism has been generally demonstrated for bacterial pathogens. One example of a bacteria modulated by SIg is *Helicobacter pylori* (*H. pylori*), a common pathogen that causes gastric mucosal inflammation, gastric cancers and peptic ulcers [68]. Indications that naturally produced SIg modulate *H. pylori* infection come from studies in pIgR KO mice [69]. These mice exhibit enhanced susceptibility to *H. pylori* infection, increased weight loss and delayed clearance compared to WT C57BL/6. Furthermore, intestinal IgA concentrations inversely correlated with *H. pylori* gastric viral load in C57BL/6 mice. A potential mechanism for the pIgR/SIg-mediated control of *H. pylori* infection was suggested by in vitro experiments, which demonstrated that human colostrum SIgA inhibited bacterial binding to human stomach tissue sections in a glycan-dependent manner [70]. The protective effect of this process in the human host is unclear since *H. pylori*-infected human gastric mucosa samples show increased levels of SC and IgA [71,72]. Thus, given the ability by *H. pylori* to establish an infection, these findings suggest that pre-existing SC and SIg were unable to prevent infection. However, whether SC and antigen-specific SIg modulate the level of *H. pylori* infection, remains to be resolved. 

Immune exclusion and host protection mediated by pathogen-specific SIg was directly demonstrated for several bacterial pathogens. For example, *Vibrio cholerae*-specific SIgA also mediate in vivo agglutination and immune exclusion [73], and reduced diarrhea severity [74] and mortality [75] in mouse models. Another example is *Shigella flexneri*, the causative agent of dysentery. *Shigella* LPS-specific SIgA protected the intestinal epithelial barrier in rabbit ileal loops from destruction by virulent *Shigella flexneri* via trapping of the bacteria in the lumen and reducing inflammation [76]. Similar findings of SIgA- and SIgM-mediated bacterial agglutination, reduced inflammation and protection of the epithelial barrier were also made in polarized Caco-2 cell monolayers [77,78]. Consistent with immune exclusion is the finding that SIgA targeting *Chlamydia trachomatis* outer membrane protein reduces infection in vitro and in vivo when binding the antigen extra-epithelially but not intra-epithelially [79].

SC and SIg may also bind pathogens during infection of naïve individuals. This scavenger function is mediated through glycosylation of SC and Ig molecules. For example, SC, through non-specific glycan interactions, agglutinates and neutralizes *Clostridium difficile* toxin A [27,80]. In addition, the glycan binding capabilities of enteropathogenic *Escherichia coli* intimin protein and type 1 fimbral lectin mediate SIgA binding, which in turn agglutinates the bacteria and prevents epithelial cell damage in vitro [81]. Natural, non-specific SIgA also reduces *Vibrio cholerae* bacterial loads in vivo, and inhibits biofilm formation in vitro [82]. Inhibition of biofilm formation is dependent on mannose-containing oligosaccharides present on SC. Natural SIgA further mediates in vivo agglutination and intra-lumenal immune exclusion of *Enterococcus faecium* [83], and *Salmonella enterica typhimurium* resulting in reduced infection and inflammation of both pathogens [84].

Taken together, these examples indicate that SC, natural SIg, and pathogen-specific SIg can mediate immune exclusion of mucosal pathogens and protect the host by a combination of innate and adaptive mechanisms. While the listed examples for immune exclusion are for bacterial pathogens, the same mechanism can be envisioned for other microbes. In fact, the microbial-scavenger function of SIgA also extends to commensal bacterial strains [26]. However, it is not universal for all microbes. SC did not bind to three rotavirus strains and in vitro infection was not impacted [80]. In addition, although murine norovirus bound to recombinant SC, non-antigen-specific SIgA did not block virus infection in cell culture or alter binding to the follicle-associated epithelium [53]. Thus, it will be of interest in the future to determine whether other viral or fungal mucosal infections can be controlled by SIg-mediated agglutination.

## 7. Intracellular Neutralization and Excretion of Pathogens

In addition to protecting the epithelial surface via extracellular complex formation, SIgA may also neutralize pathogens intracellularly while being transcytosed to the apical surface. For example, anti-Sendai virus or anti-influenza hemagglutinin specific IgA supplied to the basolateral side of polarized MCDK cells expressing pIgR was able to reduce virus infection from the apical side [85,86]. Immunofluorescence analysis demonstrated intracellular co-localization of virus and IgA, suggesting neutralization occurred inside cells. Neutralization was not observed for IgG, indicating a role for pIgR-mediated transcytosis of IgA. Similar findings were obtained with measles virus, HIV, and rotavirus [87,88,89,90]. In vitro studies of measles virus and HIV in epithelial monolayers further showed that antigen-specific IgA can bind virus on the basolateral side and mediate excretion of the immune complexes via pIgR through basolateral to apical transcytosis [87,91]. These data suggest the potential for IgA to trap pathogens that have breached the epithelial barrier and expel them from the mucosal lamina propria. 

Whether these principles extend to events in vivo has not been investigated in depth. Consistent with intracellular inactivation of virus by SIgA in vivo is a set of studies of rotavirus infection in mice [90,92]. Using a murine hybridoma backpack tumor model, which secretes a given monoclonal antibody onto mucosal surfaces via the normal epithelial transport pathway, the authors showed that rotavirus-specific IgA antibodies inhibited primary infections, resolved chronic rotavirus infections, and protected newborn mice from diarrhea upon oral challenge. Inhibition of rotavirus infection in this model was not observed with non-antigen-specific SIgA, anti-rotavirus IgG, or when antibodies were delivered directly into the intestinal lumen. Studies from knock-out mice suggest that non-antigen-specific, natural SIgA and J chain-mediated transcytosis play some role during rotavirus infection, since naïve J chain-deficient mice lacking SIg exhibited enhanced shedding and delayed clearance of rotavirus as compared to wild-type mice [93]. 

Taken together, these studies implicate intracellular neutralization by SIgA and basolateral to apical excretion of SIgA-immune complexes as a potential mechanism for protection of mucosal surfaces from viral infection. Whether these mechanisms apply broadly to all viruses infecting via mucosal surfaces or to other non-viral pathogens, or play a major role in vivo remains to be determined in future studies.

## 8. SIg-Mediated Immune Modulation during Infection 

SIgs bind pathogens either specifically through their antigen-binding domain, or non-specifically via carbohydrate residues. The fate of immune complexes in the lumen is not only restricted to immune exclusion, SIgA-immune complexes are also sampled by the host and contribute to maintaining homeostasis of the mucosa. ‘Retrotranscytosis’ (apical to basolateral) of SIgA complexes across the epithelial barrier is mediated by microfold (M) cells located in the mucosal-associated lymphoid tissue [5]. Although the identity of the receptor that mediates SIgA transcytosis on M cells remains unknown, the asialoglycoprotein receptor (ASGPR), a lectin-like receptor, or FcαRI (CD89) were ruled out as candidates [5]. Upon internalization through the M cell, “tolerogenic” DCs immediately underlying the M cell phagocytose SIgA-immune complexes [94,95]. Although binding to CD4+ T cells was noted, complexes were not internalized. Uptake of SIgA-immune complexes by mouse or human DCs is mediated via specific intercellular adhesion molecule-3 grabbing non-integrin receptor (SIGNR) 1 or the human homolog DC-SIGN, respectively [96,97]. SIgA-immune complexes can further bind to murine intestinal DCs via Dectin-1, and SIGNR3 [98]. Uptake of SIgA alone or SIgA-immune complexes by DCs is critical for dampening inflammatory immune responses in the intestinal mucosa and in turn intestinal homeostasis. For example, SIgA-primed DCs exhibited reduced DC maturation and inflammatory cytokine secretion upon TLR stimulation compared to untreated DCs [96]. These DCs further induced the expansion of Foxp3+ regulatory T cells via IL-10 and TGF-β secretion in vitro and in vivo [96]. Furthermore, *Shigella flexneri*:SIgA immune complexes reduced expression of pro-inflammatory molecules by DCs and epithelial monolayers in vitro compared to the bacterium alone [77,98]. Importantly, Peyer’s patches exposed to *Shigella flexneri*:SIgA immune complexes showed reduced induction of inflammatory mediators and tissue damage as compared to bacteria alone [76]. The anti-inflammatory effect appears to be specific to SIgA, as serum IgA-immune complexes enhance production of pro-inflammatory cytokines by monocytes and macrophages [99]. These data highlight the important role of SIgA in directly downregulating immune responses in the intestinal mucosa, thereby contributing to mucosal homeostasis.

## 9. SIg-Induced Complement Activation and Immune Pathology

Complement activation and antibody-mediated phagocytosis are important effector functions of all antibodies [100]. Therefore, SIgs may also protect the mucosal surface SIgA by these effector mechanisms. Consistent with such effector functions, SIgA agglutinates *Streptococcus pneumoniae* and opsonizes the bacteria in a complement-dependent manner [22]. However, the importance of these host defenses during bacterial pathogenesis remain to be determined. Similarly, the ability of human SIgM to activate human complement was recently demonstrated [101]. It will now be interesting to see whether SIgM mediates efficient opsonophagocytosis of pathogens at mucosal surfaces and any inflammatory consequences that might result from complement activation.

Thus, the possibility exists that SIg is not always protective but may instead contribute to disease under some circumstances. One example for the detrimental effects of SIgs comes from herpes simplex virus 2 (HSV-2), the common cause of genital herpes. J chain-deficient mice intravaginally infected with HSV-2 exhibited reduced vaginal symptoms (erythema, swelling, and ulceration) and hind limb paralysis, despite equivalent viral titers in the vaginal fluid compared to controls [102]. Additionally, treatment of intestinal organoid and immune cell co-cultures with uncomplexed SIgA triggers enhanced production of the pro-inflammatory cytokines interleukin 8 and tumor necrosis factor alpha, and increased mucus production and pIgR expression [103]. These responses were attenuated when SIgA was complexed with a commensal *Escherichia coli*, suggesting SIg elicits distinct immune responses upon antigen binding.

These data highlight that depending on the circumstances, SIg can be protective for the host via induction of immune tolerance or immune exclusion, or it can also have negative consequences for the host through activation of complement or immune-mediated histopathology.

## 10. Subversion of the pIgR/SIg System by Pathogens

Given the critical defensive role of pIgR and SIg, some pathogens have evolved strategies to hijack this system to enhance their own infection. Chiefly among those is *Streptococcus pneumoniae* (*S. pneumoniae*)—a gram-positive bacterium and a leading cause of invasive disease in children and adults worldwide [104]. *S. pneumoniae* binds to human SC [105]. Binding to pIgR aids in attachment and infection of human nasopharyngeal epithelial cells in vitro by reverse transcytosis [106]. Sensing of the infection by the host cells mobilizes intracellular calcium stores and reduces *S. pneumoniae* internalization in vitro [107]. Lack of SC in both pIgR KO and p62^yes^KO mice resulted in reduced *S. pneumoniae* lung infection [106]. Thus, high expression of pIgR in the nasopharynx is thought to promote *S. pneumoniae* colonization of the upper respiratory tract [106]. Antigen-specific SIgA is further important in protecting the host from nasal colonization [108]. Consistent with the immunologic upregulation of *PIGR*, overexpression of pIgR was observed in a mouse model of chronically inflamed lungs (i.e., SPC-HAxTCR-HA mice) [109]. However, chronic inflammation resulted in resistance rather than susceptibility to infection by *S. pneumonia*, likely because of increased levels of airway mucosal SIgA or SIgM. PIgR may also aid in *S. pneumoniae* meningitis, as pIgR was found to colocalize with *S. pneumoniae* in brain samples from human patients who had succumbed to meningitis, and anti-pIgR antibodies administered intravenously prior to infection prevented pneumococcal entry into the brain and subsequent meningitis in mice [110]. Thus, the capability of *S. pneumonia* to bind pIgR is a virulence determinant.

Another pathogen that binds to SC is *Candida albicans*—an opportunistic pathogen and important cause of vaginal infections [111]. Earlier work showed that *C. albicans* attachment to epithelial cells in vitro is aided by a component of human saliva [112]. Recently, *C. albicans* cells were demonstrated to specifically bind to free SC in saliva, and this interaction aids in epithelial cell attachment in vitro [113]. However, whether SC also aids in *C. albicans* internalization and/or infection remains to be determined.

Epstein–Barr virus (EBV)—the causative agent of infectious mononucleosis [114]—successfully hijacks the immune defense function of SIgA to expand its cell tropism. Specifically, EBV:EBV-specific SIgA immune complexes bind to pIgR on non-susceptible epithelial cells and are internalized to initiate infections in vitro [115,116]. Subsequent in vivo experiments showed that pIgR-mediated the transcytosis of EBV immune complexes via hepatocytes and aided in dissemination [117]. The ability of EBV immune complexes to be translocated from the basal to the apical side without infection was confirmed in vitro in polarized pIgR-expressing MDCK cells. In contrast, infection was observed when the cells remained unpolarized, suggesting loss of polarization predisposes epithelial cells to EBV infection following reactivation from latency in the presence of anti-EBV-specific SIgA.

In contrast to hijacking the pIgR/SIg system for their own benefit, some pathogens appear to evade the anti-microbial function by suppressing pIgR. For example, enterotoxigenic *E. coli* suppresses pIgR mRNA expression in vivo [118]. Similarly, simian immunodeficiency virus (SIV) and chimeric simian/human immunodeficiency virus (S/HIV) was able to downregulate pIgR mRNA expression in the gastrointestinal and respiratory mucosa of infected rhesus macaques [119,120], suggesting HIV may also use pIgR downregulation as an immune evasion tactic.

Taken together, these examples suggest that some bacterial, viral and fungal pathogens can subvert the protective functions of pIgR and SIg to facilitate their own infections or inhibit their defense response. It will be interesting to see in the future whether additional mucosal pathogens have evolved similar or different pIgR/SIg subversion or evasion mechanisms.

## 11. Conclusions

Taken together, overwhelming evidence supports that SC, natural SIg, and pathogen-specific SIg binding is an essential host defense mechanism aiding in pathogen exclusion, neutralization, and complement recognition. Furthermore, SIg can also regulate host immunity and mucosal tolerance via downregulation of inflammatory cytokines and initiation of regulatory immune cells. However, under some circumstances, SIg may also mediate detrimental effects for the host by inducing immune pathology. The microbiome is a critical regulator of pIgR and SIg expression, which in turn then modulates the microbiome. Although much is known regarding the microbial scavenger and immune modulatory functions of SIg, studies that address other aspects of enteric pathogen modulation and utilization of the pIgR/SIg system are less explored. While many of these outstanding questions have been detailed in the text, we have summarized them below (see Section 12). Addressing these and other future studies on how pathogens subvert the pIgR/SIg cycle will aid in further dissecting the complex roles of SIg in mucosal defense and infection. These future efforts will undoubtedly be supported by the recent developments in organoid technology [121,122] and ongoing developments that aim to incorporate additional cell types, such as immune cells [123], and link multiple organs [124]. 

## 12. Outstanding Questions

- Are pathogenic infections modulated by natural non-specific SIg or SC and do pathogens modulate that response?
- Can non-bacterial mucosal infections be controlled by SIg-mediated agglutination?
- Does intracellular neutralization of viral infections by SIgA and basolateral to apical excretion of SIgA-immune complexes extend to non-viral infections, occur in vivo, and affect within or between host spread?
- What role does SIg-induced complement activation play during infection with mucosal pathogens and colonization of commensals?
- How common are pIgR/SIg subversion or evasion mechanisms among mucosal pathogens from different kingdoms and are the strategies shared or specific?
- What is the role of SIgM-immune complexes during mucosal homeostasis and pathogenesis?
- What is the identity of the SIg receptor on M cells?
- What breakthroughs will the future hold when organoid technology is applied to the study of the pIgR/SIg cycle?
